# Severe Bushmaster Snakebite Envenoming: Case Report and Overview

**DOI:** 10.3390/reports7030068

**Published:** 2024-08-07

**Authors:** Allan Quadros Garcês-Filho, Humberto H. M. Santos, Thays K. P. P. Aguiar, Dafnin L. S. Ramos, Luis E. B. Galan, Domingos S. M. Dantas, Felipe A. Cerni, Roberto C. Carbonell, Manuela B. Pucca

**Affiliations:** 1 Medical School, Federal University of Roraima, Boa Vista 69300-000, Roraima, Brazil; allanquadros.ufrr@gmail.com (A.Q.G.-F.); hmsmachado.med@gmail.com (H.H.M.S.); thays07prado@gmail.com (T.K.P.P.A.); dafnin.lima@gmail.com (D.L.S.R.); luisbermejog@hotmail.com (L.E.B.G.); felipe_cerni@hotmail.com (F.A.C.); rcccarbonell@yahoo.es (R.C.C.); 2 Programa Doutoral de Bioética, Faculdade de Medicina do Porto, 4200-319 Porto, Portugal; saviojuazeiro@yahoo.com.br; 3 Graduate Program in Tropical Medicine (PPGMT), State University of Amazonas, Manaus 69065-001, Amazonas, Brazil; 4 Department of Clinical Analysis, School of Pharmaceutical Sciences, São Paulo State University (UNESP), Campus Araraquara, Araraquara 17033-360, São Paulo, Brazil

**Keywords:** snakebite envenoming, *Lachesis*, Roraima, infection, amputation

## Abstract

Unlike the well-documented bothropic and crotalid snakebites in Brazil, lachetic envenomings (i.e., triggered by the bushmaster snake) are rare and present significant diagnostic challenges. This case describes a severe envenoming induced by a *Lachesis muta* snake in a 26-year-old Brazilian man who was bitten near a forest in November 2022. Characteristic symptoms such as sweating and bradycardia pointed towards lachetic envenoming, but initial misdiagnosis as a bothropic bite resulted in a delay in appropriate antivenom therapy. Despite later receiving the correct treatment, the severity of the envenoming necessitated the amputation of a finger and triggered a severe infection. This report highlights the challenges of diagnosing and treating *Lachesis* spp. bites due to their rarity. Moreover, an overview of lachetic-induced signs and symptoms was explored. This study emphasizes that further reports are warranted to improve understanding of *Lachesis muta* envenoming and to optimize treatment strategies.

## 1. Introduction

Snakebite accidents represent a critical public health concern and are classified by the World Health Organization as a category A neglected tropical disease [1]. Indeed, an estimated 5.4 million cases of snakebite accidents occur worldwide annually, with 2.7 million of these cases involving envenoming, leading to up to 140,000 deaths per year. Additionally, individuals affected by snakebite accidents face a threefold higher risk of experiencing amputations or other debilitating sequelae [2].

In Brazil, there were over 180,000 reported cases of snakebite accidents between 2017 and 2022. Among these cases, approximately 70% were attributed to the *Bothrops* genus (lancehead pit vipers), while 8.5% were associated with the *Crotalus* genus (rattlesnakes). Incidents involving the *Lachesis* genus (bushmasters) accounted for 1.5%, and roughly 1% were linked to the *Micrurus* genus (coral snakes). Notably, in about 19% of cases, the genus was not specified at the time of notification. Roraima, a state situated in the northern region of Brazil, bordering Venezuela and Guyana, records the highest incidence of snakebite accidents in the country. In this region, the patterns align with national data, where snakebite accidents mainly involve *Bothrops* species, followed by occurrences related to *Crotalus* sp. Incidents involving Elapid and *Lachetic* species are less frequent [3].

**Figure 1:**
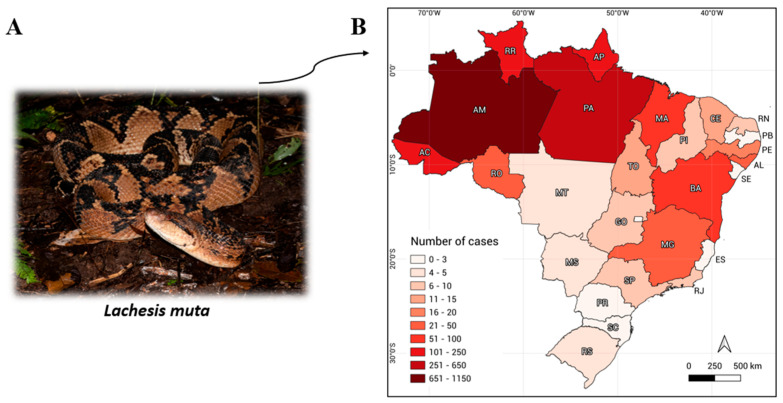
(**A**) *Lachesis muta*: the bushmaster Brazilian snake (photo by Marlus Rafael Almeida). (**B**) Number of reported bushmaster snakebites by state in Brazil from 2017 to 2022 [3].

Although rare, bushmaster snakebites pose significant danger due to their local and systemic effects. Moreover, these incidents are challenging to diagnose and treat, as medical teams seldom encounter such cases in their routine practice, resulting in underreporting. The *Lachesis muta*, the Brazilian bushmaster species, popularly known as “*surucucu pico-de-jaca*”, stands as the sole member of the Lachesis genus in Brazil. Originating from Latin, the term “muta” denotes “mute,” alluding to the striking resemblance of this species to the rattlesnake (*Crotalus durissus*) but noticeably lacking a rattle at the tip of its tail [4]. It is the largest venomous snake in Brazil, capable of reaching lengths of up to 4.5 m. *L. muta* exhibits dark spots on their heads along with a prominent post-ocular stripe, a yellowish hue with dark patterns, a white or ivory underbelly, and raised scales at the tail’s end, colored in a dark pale shade [5,6] (Figure 1A). Moreover, it inhabits most of the country, with documented accidents in the Atlantic Forest and Amazon Rainforest. Particularly noteworthy are those in the states of Amazonas (AM), Roraima (RR), Pará (PA), and Acre (AC), and in neighboring countries such as Guyana and Venezuela [3,7] (Figure 1B).

As a serpent dwelling in dense forests, bites from *L. muta* are rare due to the limited human presence in its habitat. The venom of *L. muta* exhibits proteolytic, coagulant, hemorrhagic, and neurotoxic properties, potentially resulting in compartment syndrome [8]. Therefore, the Lachesis’ snakebites are always categorized as moderate or severe, determined by the severity of symptoms and the presence of vagal manifestations (such as diarrhea, shock, bradycardia, blurred vision, and hypotension), which are not commonly observed in other snakebites across the Americas [5,8,9]. Given the venom’s impact and the usual geographical location of the incident, immediate treatment is crucial. The treatment involves administering bothropic-lachetic antivenom, as the specific lachetic antivenom has been unavailable since 1986. Treatment also includes measures aimed at stabilizing the patient [10]. This case report aims to document and review a lachetic snakebite that occurred in 2022 on the border between the state of Roraima and Guyana.

## 2. Case Presentation 

A 26-year-old indigenous man from British Guyana, residing in Brazil and previously in good health, experienced a snakebite on his left ring finger while working in the garden, next to the forest, at around 11:00 a.m. on 9 November 2022 (day 0). He encountered the snake when lifting a manhole cover, resulting in a bite on the fourth finger of his left hand. The snake was identified as a member of the *Lachesis genus*, commonly known as “*surucucu pico-de-jaca*” in Brazil. In addition to the patient’s description of the snake, clinical assessment distinguished it from *Bothrops* due to accompanying symptoms such as sweating, bradycardia, nausea, and visual disturbances, indicating vagal involvement.

**Figure 2:**
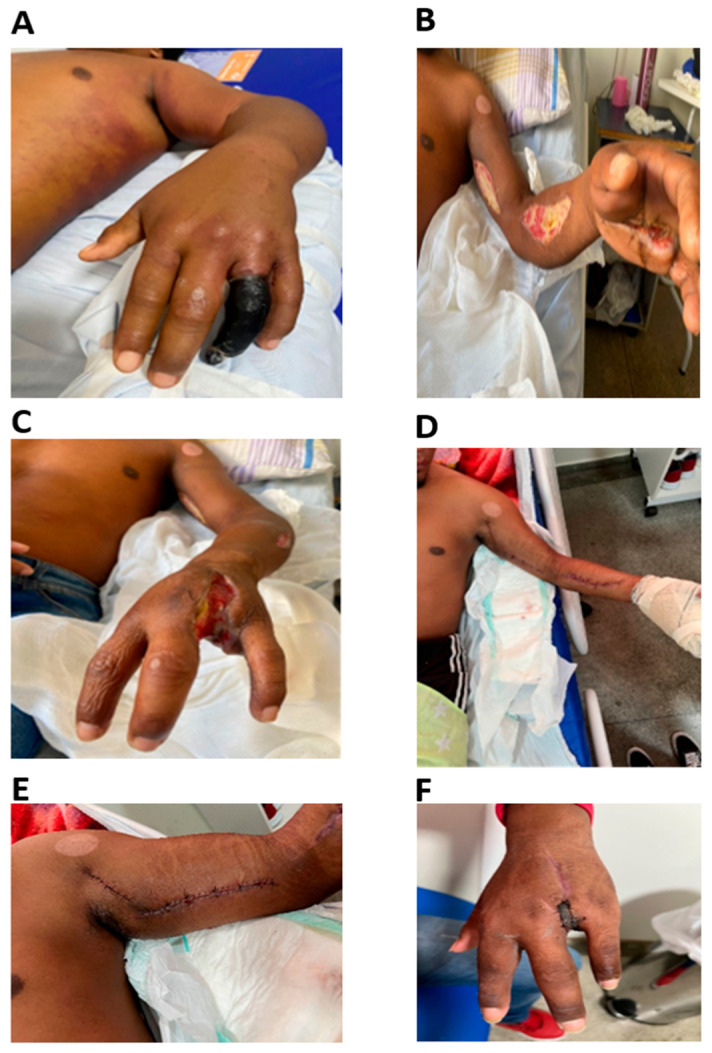
(**A**) Lachetic snakebite patient with necrosis in the 4th finger prior to amputation on 11 November (day 2, 48 h after accident). (**B**,**C**) Patient post-amputation of the 4th finger with left arm and forearm fasciotomy. (**D**,**E**) Patient post-amputation of the 4th finger with dressing and sutures at the fasciotomy sites. (**F**) Two days post-graft (day 48).

Initially, the patient received five ampoules of anti-bothropic-lachetic serum at a health center in the Uiramutã region before being transferred to the Hospital Geral de Roraima Rubens de Souza Bento (HGR). He arrived at the hospital roughly 24 h after the incident. During examination, the patient remained generally stable but complained of severe pain, significant edema (+++/4), and ecchymosis extending from the entire left upper limb to the left hemithorax and the upper left quadrant of the abdomen. Consequently, the affected finger progressed to necrosis, as noted by evaluations from the vascular surgeon and infectious-disease specialist (Figure 2A).

The treatment plan included premedication, consultation with a vascular surgeon, and a prescription for antivenom (15 ampoules of anti-bothropic-lachetic serum), completing a 20-ampoule course intended for severe lachetic envenoming. Laboratory findings revealed leukocytosis with neutrophilia and elevated serum levels of creatinine, urea, and creatine phosphokinase. The patient underwent an extensive range of tests, encompassing a complete blood count; a comprehensive biochemical profile covering parameters such as liver enzymes (ALT, AST, and ALP), total proteins, albumin, globulin, and both conjugated and unconjugated bilirubin levels; and urinalysis (Table 1).

On 11 November (day 1), a 10-day course of Ampicillin + Sulbactam antibiotics was prescribed following initial examination by the vascular surgeon. Due to the clinical deterioration observed, both a fasciotomy and the amputation of the affected finger were conducted on the same day (Figure 2B,C). An unstable skin infection detected during the surgery progressed to sepsis, leading to the patient’s transfer to the ICU for an approximate 18-day stay.

During the ICU stay, the patient experienced asymmetrical pulmonary congestion on the right side and a decreased appetite, necessitating intubation and causing subsequent challenges in ventilator weaning. However, strategic interventions focused on achieving a negative fluid balance and managing tracheal secretions resulted in notable clinical improvement, culminating in successful extubation on 26 November (day 17). Regarding the use of antibiotics, starting from day 7, Vancomycin 1 g was administered for 17 days, followed by Meropenem 1 g for 10 days. The patient was discharged from the ICU on 28 November (day 19) and transferred to the infectology and tropical medicine sector in a stable condition, devoid of sepsis signs. Encouraging advancements were observed in wound healing, with daily dressing changes involving the application of collagenase.

Between 13 December (day 34) and 25 December, the patient received care at CASAI (Indigenous Health House). Subsequently, readmission on 25 December (day 46) was necessary for reconstructive surgery, which involved a lateral surgical flap on the left arm and skin grafting for the hand and the previously affected finger due to necrosis. This surgical procedure was performed by the plastic surgery department on 27 December 2022 (day 48). The procedure included debridement and skin grafting at the site of the amputated finger (Figure 2D,E).

The patient showed favorable clinical progress following the grafting surgery, returning to the care of CASAI on the same day (day 48) (Figure 2F). Unfortunately, the current status of the patient remains unknown, as he did not attend scheduled follow-up outpatient consultations. It is presumed that approximately 60 days after the incident, the patient returned to his hometown in Guyana.

## 3. Discussion

In Brazil, four types of venomous snakes account for the majority of envenomings [10]. Among these, the genus *Lachesis* spp., specifically the species *Lachesis muta*, stands as the only representative in the country. Its subspecies, *Lachesis muta muta* and *Lachesis muta rhombeata* [11], are relatively uncommon to cause accidents due to their preference for dense forests, limiting their exposure to the general population. Despite their lower prevalence, as highlighted in the case report, incidents involving these snakes can result in severe complications and long-term consequences for the victims, given that the snake can inject between 200 and 400 mg of venom [12].

The most prevalent snake genus responsible for snakebites in the country is *Bothrops* sp., commonly known as the “*jararaca*” [10]. This type of snakebite induces various effects, including local manifestations at the bite site, such as swelling, pain, heat, and necrosis, along with systemic impacts such as coagulopathies that lead to hemodynamic disturbances, widespread bleeding, and potential complications such as acute kidney injury or shock [13,14,15,16]. The effects produced by this snake genus closely resemble those caused by snakes of the *Lachesis* genus. Consequently, diagnosing the snake responsible for the envenoming in clinical settings in the country often leads to confusion, as highlighted in Sachett et al. (2022) where a lachetic accident was mistakenly diagnosed as a bothropic one, resulting in delayed treatment for the patient [16]. Moreover, as outlined by Siva et al. (2019) in certain regions of Brazil, the local population refers to Bothrops species as “*surucucu*”, a term commonly used for Lachesis, further complicating the clinical assessment for medical professionals [4]. However, our patient, having been referred to a specialized unit in tropical medicine and infectious diseases, did not encounter a similar issue. The medical team swiftly recognized signs of vagal syndrome, a crucial means to distinguish between lachetic and bothropic accidents [16].

The complete composition of *Lachesis muta* venom remains elusive, primarily due to limited interaction between researchers and this species in the country, which makes maintaining them in captivity and obtaining venom samples in laboratories a challenging task [17]. Nevertheless, the main compounds identified, as reported by Canãs et al. (2023) include bradykinin-potentiating peptide (BPP), snake venom metalloproteinases (SVMPs), snake venom C-type lectins (SVCTLs), phospholipase A2s (PLA2s), L-amino acid oxidase (LAAO), snake venom serine proteinases (SVSPs) [17], and the recently discovered hyaluronidase and phospholipase B [11].

The local proteolytic effects are primarily triggered by metalloproteinases, as described for LHF II toxin. Although LHF-II exhibits only weak hemorrhagic and myotoxic activities, it is the primary enzyme responsible for necrosis in snakebite incidents, inducing processes such as apoptosis, necrosis, and autophagy [12,18,19]. Due to the direct effect of the snake enzyme, the cells closest to the bite site are digested and undergo cell death. The surrounding cells, however, are influenced by signals from the damaged cells, leading to a process of signal-induced apoptosis [11,12,19]. In addition to the effects of metalloproteinases, PLA2s, which are myotoxic enzymes, cause significant damage at the bite site, while LAAO has a pronounced local cytotoxic effect [18,19]. The hemodynamic effects are attributed to the thrombin-like enzyme, a serine protease [20]. 

However, the vagal effects remain poorly elucidated due to variations in intraspecific venom composition, though it is believed that bradykinins and acidic kininogenase (a serine protease) might contribute [8]. The intraspecific variation in *Lachesis muta* venom composition not only complicates its investigation but also hints at ongoing evolutionary changes within the species. This evolution could potentially pose a challenge to the efficacy of the anti-lachetic serum (i.e, antivenom) currently used in the healthcare system [21].

**Figure 3:**
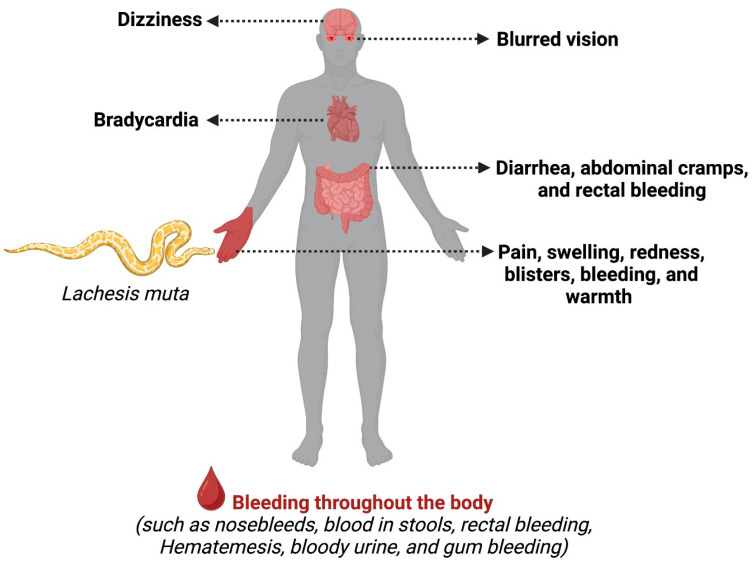
Usual signs and symptoms triggered by *Lachesis muta* envenoming. A key distinction between *Lachesis muta* envenoming and bites from other venomous snakes is vagal syndrome. This syndrome primarily manifests as sweating, bradycardia, hypotension, blurred vision, and diarrhea. Additionally, local signs at the bite site are prominent. These include inflammatory signs such as erythema (redness), pain, edema (swelling), and warmth. In severe cases, necrosis (tissue death) can occur, potentially necessitating limb amputation.

Traditionally, a patient bitten by a *Lachesis muta* snake exhibits both local and systemic symptoms (Figure 3). Among the typical local symptoms are swelling, redness, pain, blisters, bleeding, and warmth [5,8,16]. Concerning systemic symptoms, the lachetic accident may result in coagulopathy due to a disseminated intravascular coagulation, causing bleeding throughout the body (such as nosebleeds, blood in stools, rectal bleeding, vomiting blood, bloody urine, and gum bleeding) [17,22,23]. Additionally, distinctive vagal signs differentiate it from a bothropic accident: hypotension, blurred vision, severe abdominal pain/diarrhea, bradycardia, sweating, and shock [7,8,21]. Potential complications in patients include severe bleeding, acute kidney failure, compartment syndrome, necrosis, and the possibility of amputation [7].

Our patient displayed a classic case of a lachetic accident, exhibiting both local and systemic signs and symptoms. Locally, there was evident swelling, heat, redness, a foul odor, intense pain, and tissue necrosis. Remarkably, within just 24 h, the patient’s fourth finger rapidly deteriorated, leading to its amputation. Complicating the necrosis was a severe secondary infection that required intensive care unit admission for treatment with potent broad-spectrum antibiotics. It is important to note that, even with the administration of antivenom, the local damage may persist depending on the severity of the injury and the time taken to access the antivenom [12].

The loss of a limb significantly impacts an individual’s ability to work and their productivity, self-esteem, and overall quality of life. This burden is especially challenging for certain indigenous ethnicities, where amputated individuals face community rejection. Furthermore, within these communities, there exists a belief in the separation of the soul and body, which could lead to potential psychiatric implications [24,25].

Concerning systemic signs and symptoms, our analysis of laboratory tests (Table 1) revealed a marked leukocytosis primarily driven by neutrophils. This pointed towards the patient undergoing a significant inflammatory response and the potential onset of a secondary infection. These findings were consistent with the study by Elbey et al. (2017) involving 107 participants, indicating that victims of snakebites with a notably increased neutrophil-to-leukocyte ratio tend to experience prolonged hospitalizations and a higher risk of complications [26].

Furthermore, there was a decline in platelet levels within the initial 48 h, along with a slight prolongation of prothrombin time on the 11th day, suggesting a possible mild coagulopathy. However, this condition was reversed following the administration of antivenom. Additionally, within the first 48 h, the onset of acute kidney injury, possibly due to dehydration and fluid loss in the edema, was identified. This was evidenced by elevated creatinine and urea levels, which subsequently improved after stabilizing the patient’s condition and ensuring adequate hydration, corroborating the case reported by Malveira et al. (2021) [7]. Moreover, the patient exhibited signs consistent with vagal syndrome: bradycardia, sweating, nausea, and blurred vision. These vagal signs and symptoms, as highlighted by Lima and Haddad Junior (2015) and Sachett et al. (2022), assist as the primary clinical distinction between a lachetic and a bothropic accident [5,16].

There is only one recognized treatment for patients afflicted by snakebite accidents: horse-derived antivenoms. In 1986, the Ministry of Health in Brazil initiated a comprehensive national program aimed at controlling snakebites. This marked the inception of the standardization process for antivenoms [27]. In Brazil, the Ministry of Health diligently oversees the unfettered distribution of these serums to healthcare facilities across the nation, ensuring widespread access to these critical medical resources [27]. Six primary antivenoms are strategically distributed: anti-bothropic-lachetic (SABL), anti-crotalid (SAC), anti-bothropic-crotalid (SABC), anti-lachetic serum (SAL), anti-bothropic (SAB) (notably the most produced), and anti-elapidic (SAE) [28]. The anti-bothropic-lachetic serum, akin to its counterparts, is derived from hyperimmunized equines, thus triggering the production of antibodies. The immunoglobulins present in the serum play a pivotal role by binding specifically to venom-derived toxins. This targeted action facilitates neutralization of the venom, underscoring the indispensable role of antivenoms in mitigating the dire consequences of snakebites [29].

Although antivenom is the most effective way to treat a snakebite, some pre-hospital measures can reduce the morbidity and mortality associated with the bite [30]. First, ensure that the snake is no longer near the patient, move it away from the area, and do not try to capture the snake, as this poses the risk of another bite. However, it is common that victims kill the snake and bring it to the hospital, but we encourage a photo, when possible, for snake identification—the correct identification allows an accurate diagnosis of the envenoming [10,31]. Regarding wound care, it is important to check for puncture wounds or abrasions caused by the snakebite on the patient’s skin. Wash the bite area with clean water and soap when possible and monitor local and systemic signs and symptoms, such as swelling, pain intensity, and bleeding, during transport [31,32]. Finally, if possible, prevent the patient from moving and keep the affected limb in a comfortable position, preferably elevated, until arrival at the healthcare facility [10,30,31].

It is important to recognize that medications administered before hospital care do not reduce morbidity or mortality. This includes substances such as teas, herbs, and herbal concoctions such as “*Específico Pessoa*”, a traditional remedy used for treating snakebite envenomings, which lack scientific evidence of effectiveness [33]. Additionally, the use of a tourniquet, contrary to the common misconception in treating snakebite victims, is strongly discouraged. This practice can worsen localized necrosis by concentrating venom in the immobilized limb [30,31,32]. On the other hand, peptidomimetic molecules, such as batimastat and marimastat, are known to inhibit broad-spectrum matrix metalloprotease (MMP), including the SVMP-induced effects of snakebites [34]. However, to the best of our knowledge, it has not been tested for lachetic envenomings.

In cases of lachetic accidents, the primary recourse is anti-lachetic serum (SAL), or specifically, the anti-bothropic-lachetic serum (SABL) that is widely distributed in the country (Table 2). Both serums are administered intravenously. It has been demonstrated that anti-bothropic serum can be employed in confirmed lachetic accident cases, even in the absence of the specific anti-lachetic serum at the healthcare facility [17]. Nevertheless, it is substantiated that anti-bothropic serum is not capable of effectively neutralizing the proteins responsible for the anticoagulant effects induced by *Lachesis muta* envenoming [31,35].

Lachetic incidents are categorized as either moderate or severe, with the administration of 10 and 20 antivenom vials, respectively. The classification and dosage are determined based on the local and systemic manifestations presented by the patient [10]. According to the Guide for the Treatment of Snakebites (2022) from the Tropical Medicine Foundation Dr. Heitor Vieira Dourado, a case is deemed moderate when the patient displays localized symptoms, bleeding without vagal manifestations, and either a normal or altered coagulation time. On the other hand, a severe case is characterized by intense local symptoms, significant hemorrhaging, or vagal manifestations [31]. In our patient’s case, five vials of SABL were administered in the municipality of Uiramutã at the initial stage of the accident, indicating an initially ineffective treatment. Subsequently, 15 additional vials of SABL were administered, totaling the necessary 20 vials for severe lachetic incidents.

There are scarce case reports in the literature documenting lachetic accidents, particularly in recent years. Sachett and colleagues (2022) presented a case involving a 75-year-old man victim of a *Lachesis muta* snakebite [16]. It took 45 min for him to reach the hospital, displaying local bleeding accompanied by mild swelling in the bitten area. Additionally, he reported local and epigastric pain. The patient exhibited low blood pressure, sweating, tachycardia, and psychomotor agitation. Unfortunately, he received an erroneous treatment of 12 vials of bothropic antivenom. Only on the 6th day, after the administration of 11 vials of anti-lachetic serum, he was discharged. In another instance, Jorge and co-authors (1997) detailed a case involving a 28-year-old man who encountered a lachetic accident while working at a zoo, bitten during the feeding of a snake. He presented with local bleeding, swelling, and pain, arriving at the hospital approximately 1 h after the bite. The patient experienced dry mouth, abdominal pain, skin and mucosal paleness, drowsiness, sweating, nausea, vomiting, and diarrhea, with no discernible changes in vital signs. The patient received eight vials (moderate) of anti-lachetic serum 2 h after the accident; unfortunately, the report did not specify the discharge date [8]. Lastly, Pardal and colleagues (2007) documented the case of a 19-year-old man who was bitten while feeding a *Lachesis muta muta* in a zoological and botanical park. He reached the hospital 25 min after the accident, presenting with local pain, localized swelling, and bleeding at the bite site. The patient reported nausea and vomiting accompanied by paroxysmal abdominal cramps, sweating, and slight epistaxis. Vital signs indicated a slightly elevated blood pressure and tachycardia. The patient received 10 vials (moderate) of anti-bothropic-lachetic serum and was discharged three days after serum therapy [23].

The presence of a myriad of signs and symptoms in lachetic accidents is evident. While some symptoms are individualized and tailored to each patient’s unique characteristics, others form a standardized cluster observed in bothropic and lachetic accidents, including local pain, swelling, and bleeding. This complexity makes the diagnosis challenging when relying solely on local analysis. Notably, specific elements of the vagal syndrome, such as sweating, hypotension, nausea, abdominal pain, vomiting, bradycardia, and visual disturbances, are consistently present in all four cases examined, including the one detailed in this report. Consequently, scrutiny of these cases reinforces the assertion that vagal symptomatology stands as a pivotal factor in distinguishing between bothropic and lachetic accidents.

## 4. Conclusions

In contrast to other reports of lachetic accidents found in the literature, our case report was unique. It marked the first documented instance of amputation due to *Lachesis muta* bite in recent years and was the case that took the longest time to be treated appropriately. This association between the waiting time for treatment and the severity of the case was notable. Furthermore, when comparing accidents caused by *Crotalus* spp. and *Bothrops* spp., there is a scarcity of scientific data related to *Lachesis* accidents. This is attributed to the rarity of such incidents, given the snake’s predominant habitat in dense forests. The complexity of understanding the composition of *Lachesis muta* venom, its intraspecific variations, and the high potential for morbidity and mortality underscore the ongoing need for in-depth research. Potential evolutionary changes within the species may pose additional challenges to the efficacy of existing antivenom serums, emphasizing the crucial importance of continually updating the therapy to ensure clinical effectiveness. It is imperative to emphasize the significance of providing adequate training for healthcare professionals to enable early diagnosis and administer appropriate antivenom treatment. This approach helps prevent permanent sequelae and potential adverse effects on the patient’s quality of life.

## Figures and Tables

**Table 1:** Lab tests during hospitalization.

Analytes *	10 November	11 November	24 November	28 November	6 December	11 December	Reference Range **
Hemoglobin ^1^	17	13.7	7.8	11.2	11.8	11.5	13.5–18.0 g/dL
Leucocytes ^1^	32,090	28,070	12,270	9720	8640	12,730	4000–10,000 cells/µL
Neutrophils ^1^	89.8%	88%	75.8%	69.2%	41.1%	39.8%	50–70%
Platelets ^1,a^	233,000	182,000	234,000	545,000	539,000	386,000	150,000–400,000/µL
PT ^2^	13.7	14.2	11.6	12.8	14.1	-	10–14 s
INR ^2^	1.01	1.08	1	1	1.07	-	0.8–1.2
Urea ^3^	58.8	117.5	29.5	26.67	37.84	36.5	16–40 mg/dL
Creatinine ^4^	2.29	2.97	1.1	0.87	1.13	1.1	0.7–1.4 mg/dL
ALT ^3^	15.95	16.5	51.9	90.3	110.2	98.7	5–48 U/mL
AST ^5^	21.2	45.4	86.6	140.6	66.1	71	5–48 U/mL
CRP ^6^	162	-	138	48	3.4	4	0.0–8.0 mg/L

* Laboratory tests collected and analyzed by the *Laboratório Central de Roraima* (LACEM-HGR). ** Reference range from the *Laboratório Central de Roraima* (LACEM-HGR), Boa Vista, Roraima, Brazil. ^a^ The laboratory rounds the platelet results to eliminate decimal places for clarity and consistency. ^1^ Automated analysis (SF CUBE/BC-6200™, Mindray, Shenzhen, China); ^2^ automated analysis (Destiny Plus™); ^3^ kinetic-UV method; ^4^ kinetic alkaline picrate method; ^5^ enzymatic colorimetric method (Trinder); ^6^ immunoturbidimetry. ALT: alanine aminotransferase; AST: aspartate aminotransferase; CRP: C-reactive protein; PT: prothrombin time; INR: international normalized ratio.

**Table 2:** Differences in clinical manifestations, classification, type, and number of vials used in lachetic versus bothropic envenomings.

Snake	Clinical Manifestation	Type of Serum	Number of Ampoules
*Bothrops* spp.	Mild: The most common form of envenoming, characterized by mild or no local pain and swelling and mild or absent hemorrhagic manifestations.		2–4
	Moderate: Characterized by pain and significant swelling that extends beyond the bitten anatomical segment. Moderate envenoming may or may not be accompanied by local or systemic hemorrhagic alterations.	SAB; SABL; SABC	4–8
	Severe: Characterized by pain and significant swelling that extends beyond the bitten anatomical segment. Moderate envenoming may or may not be accompanied by local or systemic hemorrhagic alterations.		12
*Lachesis* spp.	Moderate: Local symptoms present, with or without bleeding. Some cases may lack vagal manifestations, making them challenging to differentiate from Bothrops envenoming.		10
		SABL	
	Severe: Intense local symptoms, severe bleeding, and/or vagal manifestations.		20

Table created using published data [10,31]. The primary difference between the two types of envenoming is the presence or absence of vagal symptoms. SAB: Bothropic antivenom, from Portuguese “*soro anti-botrópico*”. SABL: Bothropic/lachetic antivenom, from Portuguese “*soro anti-botrópico-lachetico*”. SABC: Bothropic/crotalid antivenom, from Portuguese “*soro anti-botrópico-crotálico*”.

## Data Availability

The original data presented in this study are available on reasonable request from the corresponding author. The data are not publicly available due to privacy.
